# Prediction of Streptococcus uberis clinical mastitis risk using Matrix-assisted laser desorption ionization time of flight mass spectrometry (MALDI-TOF MS) in dairy herds

**DOI:** 10.1016/j.prevetmed.2017.05.015

**Published:** 2017-09-01

**Authors:** Simon C. Archer, Andrew J. Bradley, Selin Cooper, Peers L. Davies, Martin J. Green

**Affiliations:** a University of Nottingham, School of Veterinary Medicine and Science, Sutton Bonington Campus, Sutton Bonington, Leicestershire, LE12 5RD, United Kingdom; b Quality Milk Management Services Limited, Cedar Barn, Easton, Wells, Somerset BA5 1DU, United Kingdom

**Keywords:** MALDI-TOF MS, matrix-assisted laser desorption ionization time of flight mass spectrometry, CCI, composite correlation index, CMP, potentially contagious MALDI-TOF MS profile, EMP, potentially environmental MALDI-TOF MS profile, BCI, bayesian credible interval, IQR, interquartile range, SCC, milk somatic cell count, Prediction, Contagious mastitis, *Streptococcus uberis*, Molecular epidemiology, MALDI-TOF MS

## Abstract

The purpose of this study was to evaluate whether the risk of *Streptococcus uberis* clinical mastitis at cow level could be predicted from the historical presence of specific strains of *S. uberis* on dairy farms. Matrix-assisted laser desorption ionization time of flight mass spectrometry was used to identify *S. uberis* isolates potentially capable of contagious transmission. Data were available from 10,652 cows from 52 English and Welsh dairy farms over a 14 month period, and 521 isolates of *S. uberis* from clinical mastitis cases were available for analysis. As well as the temporal herd history of clinical mastitis associated with particular *S. uberis* strains, other exposure variables included cow parity, stage of lactation, milk yield, and somatic cell count. Observations were structured longitudinally as repeated weekly measures through the study period for each cow. Data were analyzed in a Bayesian framework using multilevel logistic regression models. Similarity of mass spectral profiles between isolates of *S. uberis* from consecutive clinical cases of mastitis in herds was used to indicate potential for contagious phenotypic characteristics. Cross validation showed that new isolates with these characteristics could be identified with an accuracy of 90% based on bacterial protein mass spectral characteristics alone. The cow-level risk in any week of these *S. uberis* clinical mastitis cases increased with the presence of the same specific strains of *S. uberis* in other cows in the herd during the previous 2 weeks. The final statistical model indicated there would be a 2–3 fold increase in the risk of *S. uberis* clinical mastitis associated with particular strains if these occurred in the herd 1 and 2 weeks previously. The results suggest that specific strains of *S. uberis* may be involved with contagious transmission, and predictions based on their occurrence could be used as an early warning surveillance system to enhance the control of *S. uberis* mastitis.

## 1 Introduction

Mastitis control is of considerable importance to dairy producers in terms of potential economic, environmental, and welfare constraints (Hospido and Sonesson, 2005, Halasa et al., 2007, Kemp et al., 2008). In order to control mastitis, the risk pathways and appropriate corrective interventions need to be understood (Bramley and Dodd, 1984). For control strategies to be effective and efficient in terms of utilising resources, farm-specific intervention plans have been shown to perform well (Green et al., 2007), dependent on ongoing mastitis control efforts. An alternative approach could be to target control efforts towards high risk periods, if these could be identified in advance. This would allow herd managers to ensure control policies are effectively enhanced when most needed. New intramammary infections are thought to arise from a reservoir of pathogens either in the environment, or other cows (Leigh, 1999, Barkema et al., 2009), and for the common mastitis pathogen, *Streptococcus uberis*, transmission is thought possible through both routes (Zadoks et al., 2003, Rato et al., 2008).

The risk of *S. uberis* mastitis cases may be dependent upon the presence of specific strains of this organism carried by other cows within a farm. Multilocus sequence typing has provided insights into the epidemiology of *S. uberis* mastitis (Rato et al., 2008, Gilchrist et al., 2013, Davies et al., 2016), suggesting that although ‘environmental’ strains appear heterogeneous (Lopez-Benavides et al., 2007), clonal strains in different cows can be identified suggesting contagious transmission. To date there is no information to determine whether the presence of specific strains of *S. uberis* can be used to predict future mastitis risk on the farm. Furthermore, the time required for existing strain typing techniques implies they have not been applied for large scale diagnostics in real time, when results are required rapidly to inform decision making.

Matrix-assisted laser desorption ionization time of flight mass spectrometry (MALDI-TOF MS) relies on the differential velocity of positive ions in a vacuum based on their size and charge following ionization with a laser. It has been used in milk quality laboratories both diagnostically and in research, to facilitate bacterial species identification (Goncalves et al., 2014, Bradley et al., 2015). Elsewhere, the technique has also proved useful for the rapid strain typing of pathogenic organisms (Barbuddhe et al., 2008, Dubois et al., 2010, Rizzardi et al., 2013). The purpose of this research was to determine if MALDI-TOF MS could be used to identify potentially contagious strains of *S. uberis* and hence predict clinical mastitis risk at cow level throughout a longitudinal study in 52 commercial dairy herds, based on the observation of 10,652 cows.

## 2 Materials and methods

### 2.1 Data collection

Observational cow level data and milk samples from 3337 clinical mastitis cases collected over 14 months from March 2004 were available from a previous study on 52 dairy farms in England and Wales (Green et al., 2007). Continuous monthly records of milk somatic cell count (SCC) and yield were available from these farms. Farmers collected milk samples from clinical cases of mastitis for 12 consecutive months on each farm, commencing over a 2 month recruitment period. Samples were cultured and isolates identified using standard laboratory methods for the microbiological analysis of milk (National Mastitis Council, 1999); 1099 putative *S. uberis* isolates were stored on micro-preservation beads at −80 °C.

### 2.2 Classification of clinical mastitis cases

Eight hundred and fifty four recovered putative *S. uberis* isolates were incubated once on blood agar at 37 °C for 18–24 h. Protein extraction used ethanol to lyse cells followed by centrifugation, as has been previously described (Barreiro et al., 2010, Werner et al., 2012). MALDI-TOF MS (Bruker Daltronics, Billerica, MA) used the Bruker Library (Version 5989) to confirm 521 isolates as *S. uberis* that were suitable for inclusion based on the following criteria. Isolates required appropriate cow-level identification to link with observational data for subsequent analyses. Six replicates of protein mass spectra in the range 4000 to 10,000 Da were generated. Spectral replicates of each isolate were compared visually using Biotyper 3.1 (Bruker Daltonics, Coventry, UK); those spectra with insufficient resolution, low intensity, or substantial background noise were discarded from further analyses. At least three good quality spectra per isolate were required for inclusion. Eighteen per cent of putative *S. uberis* isolates identified biochemically, were classified as other bacteria by MALDI-TOF MS and excluded. These were predominantly *Streptococcus spp*., *Enterococcus spp*., and *Escherichia Coli*. Since many of the remaining *S. uberis* isolates had also undergone genetic typing in a separate study (Davies et al., 2016), we consider there was more error with biochemical identification than with MALDI-TOF MS, and misclassification of *S. uberis* at species level for the isolates included in the study was unlikely.

The composite correlation index (CCI) returns a number between 0 and 1 representing similarity of the available spectra from any 2 isolates, through a computation as defined by Arnold and Reilly (1998). In this research, a threshold was set such that if the CCI for any 2 spectra was at least 0.99, they would be considered the same *S. uberis* strain. Remaining spectral replicates following visual inspection were therefore compared using CCI, to remove dissimilar spectra with CCI < 0.99. Remaining spectra following this step were used in the subsequent analysis. For comparisons of isolates within and between herds, matrices based on mastitis cases by date and CCI were generated with spectra from the 521 isolates.

Previous research has indicated that transmission within and between cows may occur with a limited number of *S. uberis* strains (Zadoks et al., 2003, Zadoks et al., 2005). Therefore, spectra were grouped based on CCI values to indicate similarity or difference between isolates and hence possible contagious or environmental phenotypes as follows. *S. uberis* isolates from consecutive clinical cases, that occurred at any time in different cows within the same herd were defined as having a ‘potentially contagious MALDI-TOF MS profile’ (CMP) when the CCI was ≥ 0.99; with this degree of similarity the strains were considered to be the same. The maximum interval observed between consecutive cases was 43 weeks, although 75% of intervals were did not exceed 5 weeks. Sixty nine isolates were identified with profiles in the CMP category. From the same 32 herds in which the CMP isolates occurred, fifty four *S. uberis* isolates with spectral profiles that differed both from the CMP strains and from each other were also identified. To ensure clear differentiation from the CMP strains and adequate group size for analysis, the spectral profiles from these *S. uberis* isolates were required to have a CCI ≤ 0.96 with any other isolate in the dataset. This heterogeneous group, were defined as having an ‘environmental MALDI-TOF MS profile’ (EMP). We therefore constructed 2 groups based on the selected criteria.

Protein mass spectra from isolates in the CMP and EMP groups were compared, to evaluate how accurately the two groups could be differentiated based solely on their protein mass spectral profiles. Analysis was conducted using ClinProTools 2.2 (Bruker Daltronics) and spectral peaks were selected for comparison following a procedure to subtract baseline variation, calculated by a supervised learning based ‘Top Hat’ algorithm with similarity selection to filter within the sample replicates (Serra, 1982). A model to identify CMP from EMP isolates based on whole cell protein mass spectra was generated using a Supervised Neural Network algorithm (Hammer et al., 2005), with fit to the data compared based on internal recognition of the same isolates. To indicate predictive ability in external data not used for model development, cross validation was carried out by leaving out 20% of the data at random, re-fitting the model and predicting the omitted data; this procedure was carried out 10 times, and the results were averaged (Kearns et al., 1997). Visualisation of differences used principal component analysis with level scaling (van den Berg et al., 2006). Following training of the model with the dataset containing known CMP and EMP profiles, differences in mass spectral protein peaks characterised in the model were used to classify the other 398 *S. uberis* isolates as either CMP or EMP. This final classification was used in a statistical model to demonstrate predictive performance using observational data from all 52 herds.

### 2.3 Data preparation

A longitudinal time series was constructed, with each week of the study period listed as repeated measures for each cow in the 52 herds, giving a dataset of 474,779 rows. The outcome of interest was the occurrence at least 1 case of CMP, *S. uberis* clinical mastitis in any week for each cow. Exposure variables investigated were parity, lactation week, and data from routine monthly herd test days (milk yields, constituents and SCC). Herd level geometric mean SCC and proportions of cows with SCC > 200,000 cells/mL were calculated for each farm at each monthly recording, together with cow milk yield and SCC; these variables were split into categories based on quartiles of the data. A further category was included for each variable to indicate when data were missing, and this was used in weeks with no routine recording. Categorical variables were lagged backwards and forwards for 8 weekly periods to investigate temporal relationships with the occurrence of CMP clinical mastitis cases. At herd level, the occurrence of 1 or more CMP isolates in any cow, in any week was coded as a binary variable (present in the herd or not), and appended to the dataset. This variable was lagged backwards, allowing the occurrence of CMP cases in the herd over the previous 8 weeks to be compared prospectively to the weekly occurrence of CMP cases in individual cows.

### 2.4 Statistical model development

The binary outcome (y_ijk_) was the occurrence of one or more CMP clinical mastitis cases in the i^th^ week, j^th^ cow, and k^th^ herd. The model therefore took the form:

y_ijk_ ∼ Bernoulli (probability = π_ijk_),

logit (π_ijk_) = α + β_1_X_ijk_ + β_2_ X_jk_ + β_3_X_k_ + v_k_ + u_jk_,

v_k_ ∼ Normal (0, σ^2^_v_),

u_jk_ ∼ Normal (0, σ^2^_u_),

where, α = intercept value, X_ijk_ = matrix of exposure variables for each week of the study such as production data, β_1_ = vector of coefficients for X_ijk_, X_jk_ = matrix of exposure variables for each cow such as parity, β_2_ = vector of coefficients for X_jk_, X_k_ = matrix of exposure variables for each herd such as the occurrence of 1 or more CMP cases, β_3_ = vector of coefficients for X_k_, v_k_ = random effect to account for residual variation between herds (assumed to be a normal distribution with mean = 0 and variance σ^2^_v_), u_jk_ = random effect to account for residual variation between cows (assumed to be a normal distribution with mean 0 and variance σ^2^_u_). Models were built using a forward selection procedure, and sequentially including test variables and plausible interactions, retaining those in which the coefficient was ≥ (1.96 * standard error). Polynomial terms were evaluated for continuous variables; study week, and lactation week. Parity was investigated as categorical variables with 1–4 bins. Milk production and SCC data from monthly recordings were investigated as categorical variables based on quartiles of the data and a missing category for weeks with no recording. Estimation of initial values for parameters used an iterative generalised least squares algorithm in MLwiN (Browne, 2012). Posterior distributions for candidate model parameters were generated in a Bayesian framework using vague prior distributions for β ∼ Normal (0, 10^6^), σ^−2^_v_ ∼ Gamma (0.001, 0.001), and σ^−2^_u_ ∼ Gamma (0.001, 0.001). Ten thousand Markov chain Monte Carlo simulations were recorded after a burn in of 2000 simulations when convergence was deemed to have occurred (Browne, 2012). The fit of subsets of models were compared using Deviance Information Criteria (Spiegelhalter et al., 2002). In order to check fit to the data, the final models were transferred to WinBUGS 1.4 (Lunn et al., 2000), and posterior predictions of CMP clinical mastitis occurrence were generated for every line of the database. Distributions of predicted CMP risk at cow level were compared to the observed cow-level CMP risk stratified by categorical variables in the model such as cow parity, and time since calving. As an indicator of model usefulness (Gelman et al., 1996), predictions were repeated for categorical variables that were not in the model such as the rolling herd geometric mean SCC over the study period. For comparison, a model with the occurrence of EMP and CMP cases as a single binary outcome was created using the method described above. This was to identify whether the occurrence of CMP cases in the herd was predictive of all *S. uberis* cases, rather than only those suspected to occur as a result of contagious transmission.

## 3 Results

### 3.1 Classification of clinical mastitis cases

With the final classification for *S. uberis* clinical mastitis cases based on differences in protein mass spectra alone, internal recognition of the 69 CMP isolates from 64 cows used in model training was 97%, and for the 54 EMP isolates from 50 cows used for comparison, internal recognition was 81%. During cross validation the classification model correctly identified 90% of the CMP isolates (sensitivity), and 69% of the EMP isolates from clinical mastitis cases in these selected groups (specificity). The model was used to classify remaining *S. uberis* cases as CMP or EMP isolates for statistical analysis. Weekly clinical mastitis incidence for cases in each group stratified by parity, quarter of the year, and stage of lactation varied between herds (Table 1). The distribution of time intervals between consecutive cases of CMP mastitis cases within herds was skewed with a maximum of 43 weeks. However, the median interval (interquartile range (IQR)) between consecutive cases of CMP mastitis cases within herds was 2 weeks (IQR; 1–5), with a minimum interval of 0 weeks.

**Table 1:** Medians and interquartile ranges for stratified mean herd level weekly clinical mastitis incidence, for cases associated with *Streptococcus uberis* isolates with high and low correlation determined using Matrix-assisted laser desorption ionization time of flight mass spectrometry composite correlation indices; high correlation suggested a contagious MALDI-TOF MS profile (CMP), and low correlation suggested an environmental MALDI-TOF MS profile (EMP).

	CMP			EMP		
	Lower 25%	Median	Upper 75%	Lower 25%	Median	Upper 75%
Parity						
1	0.0000	0.0003	0.0007	0.0000	0.0000	0.0006
2	0.0000	0.0000	0.0008	0.0000	0.0005	0.0011
3	0.0000	0.0002	0.0008	0.0000	0.0000	0.0011
> = 4	0.0003	0.0006	0.0018	0.0002	0.0006	0.0014

Quarter of year						
January to March	0.0000	0.0000	0.0007	0.0000	0.0004	0.0012
April to June	0.0000	0.0004	0.0013	0.0000	0.0003	0.0007
July to September	0.0000	0.0003	0.0011	0.0000	0.0000	0.0008
October to December	0.0000	0.0007	0.0013	0.0000	0.0006	0.0016

Stage of lactation						
<91 d	0.0003	0.0007	0.0013	0.0001	0.0005	0.0010
91 to 180 d	0.0000	0.0003	0.0010	0.0000	0.0003	0.0010
181 to 270 d	0.0000	0.0000	0.0009	0.0000	0.0004	0.0011
>270 d	0.0000	0.0000	0.0000	0.0000	0.0000	0.0019

### 3.2 Descriptive results

Overall, the median herd had 12 cases of *S. uberis* clinical mastitis identified using MALDI-TOF MS (IQR; 6–19) from 202 cows at risk (IQR; 128–248). The maximum weekly incidence rate was 4 CMP and 1 EMP cases per 100 cow weeks at risk (Farm 27, week 53). The weekly incidence rate for the median, lower quartile, and upper quartile week was 0 cases per 100 cow weeks at risk. The median herd had 4 CMP *S. uberis* cases (IQR; 2–10), and 4 EMP *S. uberis* cases (IQR; 3–8). The maximum weekly CMP incidence rate was 3 cases per 100 cow weeks at risk (Farm 68, week 37), and the maximum weekly EMP incidence rate was 4 cases per 100 cow weeks at risk (Farm 27, week 52).

### 3.3 Statistical model results

The final logistic model for the occurrence of CMP *S. uberis* cases is summarized in Table 2. Cows in parities ≥4 had increased median odds of CMP clinical mastitis by a factor of 2.1 (95% Bayesian credible interval (BCI); 1.7–2.7) compared to cows in parities <4. Odds of CMP clinical mastitis in any cow increased if 1 or more CMP cases were identified in other cows in the same herd during the previous 2 weeks. The median odds of CMP mastitis in any cow each week increased by factors of 2.2 (95% BCI; 1.7–2.9), and 1.4 (95% BCI; 1.1–1.9) with CMP cases in the same herd one and 2 weeks previously, compared to no CMP cases in the herd. The adjusted influence of other factors including stage of lactation and time of year (Table 1), were found not to influence the occurrence of CMP cases with at least 95% certainty. Three cows had repeat CMP cases in consecutive weeks. Inclusion of these cases in the analysis did not influence the results. A comparative model, with all *S. uberis* cases (CMP and EMP) as a single binary outcome did not identify associations with the previous occurrence of CMP cases in the herd.

**Table 2:** Median and 95% Bayesian credible intervals for odds ratios (unless shown otherwise) from 10,000 simulations of the final logistic model for the occurrence of clinical mastitis cases at cow level in any week associated with *Streptococcus uberis* isolates with highly correlated protein spectra determined using Matrix-assisted laser desorption ionization time of flight mass spectrometry composite correlation indices (contagious MALDI-TOF MS profile (CMP)).

	Lower 2.5%	Median	Upper 97.5%
Fixed part:			
Intercept^a^	−8.69	−8.33	−8.01
Parity ≥ 4	1.71	2.14	2.67
Lactation week^b^	0.97	0.98	0.99
(Lactation week)^2	1.00	1.00	1.00
≥ 1 CMP in herd 1 week before	1.69	2.21	2.86
≥ 1 CMP in herd 2 week before	1.08	1.44	1.92
Random part^c^:			
Farm level variance	0.48	0.85	1.54
Cow level variance	0.07	0.12	0.23

a Parity 1–3, no CMP in the herd in the last 3 weeks and week 19 of lactation (logit scale).

b Quadratic term centred on the mean (week 19).

c Logit scale.

### 3.4 Statistical model predictions

Predictions of dataset strata defined by parity, lactation month, and rolling herd geometric mean SCC indicated good fit and usefulness of the final model, with observed values all within the 95% BCI of predictions. Inclusion of other variables, such as cow and herd level summaries of production, and SCC data did not improve fit to the data in either case. Relative risk predictions for forthcoming time periods are demonstrated in Table 3. The baseline median predicted risk of CMP cases was 3 per 10,000 cow weeks (IQR; 3–5; Table 1); this would at least double with occurrence of CMP cases in the herd 1 and 2 weeks previously (95% BCI for relative risk: 2.01–3.59; Table 3).

**Table 3:** Summary of model predictions for the relative cow-level risk of *Streptococcus uberis* clinical mastitis cases with contagious characteristics (CMP)^a^.

Scenario	Lower 25%	Median	Upper 75%
At least one CMP case in the herd both 1 and 2 weeks previously (compared to none)	2.81	3.18	3.59
At least one CMP case in the herd in the last week (compared to none)	2.01	2.21	2.42
At least one CMP case in the herd between 1 and 2 weeks ago (compared to none)	1.31	1.44	1.59

a Determined based on a highly correlated protein spectra using matrix-assisted laser desorption ionization time of flight mass spectrometry composite correlation indices (Biotyper 3.1; Bruker Daltronics, Coventry, UK). Predictions refer to a herd with average stage of lactation of 19 weeks, and 25% of cows in parity 4 or higher.

## 4 Discussion

This research illustrates a method to predict future high risk periods for the potential transmission of *S. uberis* clinical mastitis in dairy herds based on the occurrence of specific *S. uberis* strains during the previous 2 weeks. Importantly, this occurred preferentially for those *S. uberis* strains that were phenotypically more likely to be contagious than environmental, based on characteristic differences in their protein mass spectra. This is the first study to predict high risk periods for clinical mastitis cases in advance, enabled by MALDI-TOF MS for rapid identification of potentially contagious strains. If routinely applied, information on the presence of potentially contagious *S. uberis* strains would be available 2 weeks before high risk periods occurred, prioritising immediate and timely enhancement of appropriate preventive measures (Barkema et al., 2009). Transmission to other cows is a critical component of udder health costs (Down et al., 2013), meaning these warnings could be particularly valuable in the control of contagious disease. For instance, prediction of increased risk could be used to increase compliance with unpopular, labour intensive interventions (Huijps et al., 2009) such as post milking teat disinfection (Galton, 2004), or segregation of cows.

From genetic analyses, contagious transmission of *S. uberis* mastitis between cows is believed to be a feature of relatively few cow adapted strains that may be over-represented in cases of *S. uberis* clinical mastitis (Davies et al., 2016), compared to the heterogeneity of environmental isolates (Zadoks et al., 2003, Zadoks et al., 2005). In this research, protein mass spectral characteristics of *S. uberis* isolates with contagious phenotypes were found to differ from those with environmental phenotypes. Prediction based on the occurrence of index EMP cases in herds was not considered biologically plausible, as the epidemiology is strongly influenced by environmental conditions for which data were unavailable. Notably, the past occurrence of CMP cases in herds was only predictive of further CMP cases, and not of increased risk of any *S. uberis* isolates (CMP and EMP) in the coming weeks. Our results are therefore consistent with previous research, indicating that contagious transmission is probably a feature of a sub-set of *S. uberis* isolates (Zadoks et al., 2003, Davies et al., 2016). Identifying these characteristics based on the expression of bacterial proteins rapidly provides prospective information indicating increased potential risk of contagious *S. uberis* spread within herds.

Based on differences in protein expression alone, *S. uberis* strains with highly correlated protein mass spectra (CCI ≥ 0.99) compared with other isolates in the database, were identified with an accuracy of 90%. It is hypothesised these represent contagious *S. uberis* strains; however it should be acknowledged that a gold standard for definitive transmission of cases is unavailable. *S. uberis* strains with low correlation between protein mass spectra (CCI ≤ 0.96) compared with other isolates in the database were hypothesised to be environmental *S. uberis* isolates. These were identified with an accuracy of 69%; this was expected because some isolates could be misclassified, if they were actually contagious isolates that did not have an opportunity to spread during the study period.

Assumed transmission of CMP clinical mastitis cases in this study may not have been direct; intermediate subclinical cases or missing clinical cases could have occurred. The possibility for indirect transmission may be limited, as risk of transmission was highest when the interval from potential contagious cases in the herd was just 1 week, and declined when the interval was 2 weeks, consistent with previously observed skewed distributions for infection duration (Zadoks et al., 2003). Furthermore, no indication of associations between *S. uberis* mastitis transmission risk and SCC history were identified. Possible explanations for this are that SCC changes were not specific for *S. uberis* compared to other pathogens, or monthly cow SCC records were insufficiently sensitive to transient quarter level changes. An alternative explanation for our results is presence of supposed contagious strains in the environment, as a point source of infection. The highest observed *S. uberis* clinical mastitis risk (CMP and EMP cases; 5 cases per 100 cow weeks) in the study was comparable to that in a previously reported outbreak (Zadoks et al., 2001), suggesting that the results of this study may be generalizable. Application of the results in a predictive manner would be dependent on the availability of appropriate samples and data from clinical mastitis cases. The pay back would be in decreasing the time to adapting farm specific mastitis control plans, as risk predictions could be made from an index case, for example in the face of a contagious mastitis outbreak. The scientific findings therefore raise the possibility for the usefulness of our predictive models in future mastitis control plans. However we acknowledge that substantial further work and validation is required for development as a useful clinical service.

## 5 Conclusion

In this study, the potential risk of *S. uberis* clinical mastitis transmission increased if other contagious *S. uberis* isolates were identified previously in the same herd. Possible contagious *S. uberis* isolates can be rapidly identified using MALDI-TOF MS. This information is predictive of the risk of future related cases, and therefore potentially provides a completely novel approach for risk prediction and management of mastitis on farms.
